# Extraction of Gold(III) from Hydrochloric Acid Solutions with a PVC-based Polymer Inclusion Membrane (PIM) Containing Cyphos^®^ IL 104

**DOI:** 10.3390/membranes5040903

**Published:** 2015-12-08

**Authors:** Ya Ya Nutchapurida Bonggotgetsakul, Robert W. Cattrall, Spas D. Kolev

**Affiliations:** School of Chemistry, The University of Melbourne, Victoria 3010, Australia; E-Mails: yayabong88@gmail.com (Y.Y.N.B.); r.cattrall@unimelb.edu.au (R.W.C.)

**Keywords:** polymer inclusion membrane (PIM), extraction, Cyphos^®^ IL 104, gold recovery

## Abstract

Abstract: Poly(vinyl chloride) (PVC) based polymer inclusion membranes (PIMs), with different concentrations of Cyphos® IL 104 as the membrane extractant/carrier, were studied for their ability to extract Au(III) from hydrochloric acid solutions. Some of the PIMs also contained one of the following plasticizers or modifiers: 2-nitrophenyloctyl ether, dioctylphthalate, 1-dodecanol, 1-tetradecanol, or tri(2-ethylhexyl) phosphate. The best performance, in terms of extraction rate and amount of Au(III) extracted, was exhibited by a PIM consisting of 25 wt% Cyphos^®^ IL 104, 5 wt% 1-dodecanol, and 70 wt% PVC. An almost complete back-extraction of the Au(III) extracted from this membrane was achieved by using a 0.10 mol L^−1^ Na_2_SO_3_ receiver solution at pH 8. The stoichiometry of the extracted Au(III)/Cyphos® IL 104 adduct was determined as [P]^+^ [AuCl_4_]^−^ H^+^ [PO_2_]^−^ where [P]^+^ and [PO_2_]^−^ represent trihexyl(tetradecyl) phosphonium and bis(2,4,4-trimethylpentyl) phosphinate ions, respectively. Back-extraction of Au(III) is suggested to occur by reduction of Au(III) to Au(I), with the formation of the species [Au(SO_3_)_2_]^3−^ in the aqueous receiver solution. Loss of 1-dodecanol from the newly developed PIM to the aqueous solutions in contact with it was observed, which indicated that this membrane was suitable for single use in the efficient recovery of Au(III) from hydrochloric acid solutions of electronic scrap or recycled jewelry.

## 1. Introduction

During the past decade, there have been a number of studies of gold recovery from acid solutions using polymer inclusion membranes (PIMs) [1,2,3,4,5]. It has been reported that PIMs offer advantages over traditional solvent extraction, as they mimic the latter’s separation ability but without the use of a large inventory of diluents, which are often highly volatile and flammable liquids [6,7,8].

PIMs consist of an extractant (often referred to as carrier) and a base polymer (commonly poly(vinyl chloride) (PVC) or cellulose triacetate (CTA)). In some cases, they may also contain a plasticizer or modifier. The extractant is usually a liquid complexing agent or an ion-exchanger, responsible for binding with the target species and transporting it across the PIM; plasticizers improve the compatibility between the extractant and the base polymer, thus, making the membrane homogeneous and flexible, while modifiers enhance the solubility of the adduct of the extractant and the target species in the membrane liquid phase [7]. Several extractants have been used in Au(III) recovery from acidic solutions, such as Aliquat 336 [1,5], Kelex 100 [2], ω-thiocaprolactam [4] (this PIM was used mainly for Pd(II) separation), and thiacalix[4]arenes [3]. These studies have shown successful extraction of Au(III) from hydrochloric acid solutions, with good selectivity over other base metal ions. However, difficulties with efficient stripping of Au(III) from the proposed PIMs have been encountered, *i.e*., in the case of the Kelex 100-based PIM, stripping of up to 85% of Au(III) could be achieved [2], while up to 65% of Au(III) could be transported across a thiacalix[4]arene-based PIM [3]. Argiropoulos *et al.* [1] have reported on the high selectivity of a PIM composed of PVC and Aliquat 336 as the carrier for Au(III) extraction from hydrochloric acid solutions, even in the presence of a 500-fold higher concentration of Cu(II). However, they encountered difficulties in stripping Au(III) in the receiving solution during transport experiments, and observed some instabilities of the membrane.

Guo *et al.* [9] have reported on the successful use of a PIM incorporating the ionic liquid extractant Cyphos^®^ IL 104 as the extractant/carrier and polyvinylidene fluoride (PVDF) as the base polymer for Cr(VI) transport. The membrane showed a faster transport rate and a higher stability than that using Aliquat 336 as the carrier. The stripping of the target chemical species was easily achieved by using a NaOH solution. The present paper describes the use of a PVC-based PIM containing Cyphos^®^ IL 104 as the carrier for the efficient extraction of Au(III) from hydrochloric acid solutions.

## 2. Results and Discussion

### 2.1. Optimization of the Membrane Composition 

An initial study of the membrane composition with Cyphos^®^ IL104 as the carrier and PVC as the base polymer was carried out by preparing membranes with the following compositions: Cyphos^®^ IL 104 (20–40 wt%) and PVC (60–80 wt%) or with Cyphos^®^ IL 104 (20 wt%), PVC (70 wt%) and 10 wt% of one of the following plasticizers or modifiers: 2-nitrophenyloctyl ether, dioctylphthalate, 1-dodecanol, 1-tetradecanol, and tri(2-ethylhexyl) phosphate. It was observed that only PIMs prepared with the extractant alone, or with the addition of the modifier 1-dodecanol, were homogeneous, transparent, and flexible. The membranes containing the other plasticizers and modifiers had oily surfaces, which indicated incompatibility of their components. Therefore, membranes were prepared for further study with the compositions shown in Table 1, and the appearance of these PIMs was observed visually.

**Figure 1:**
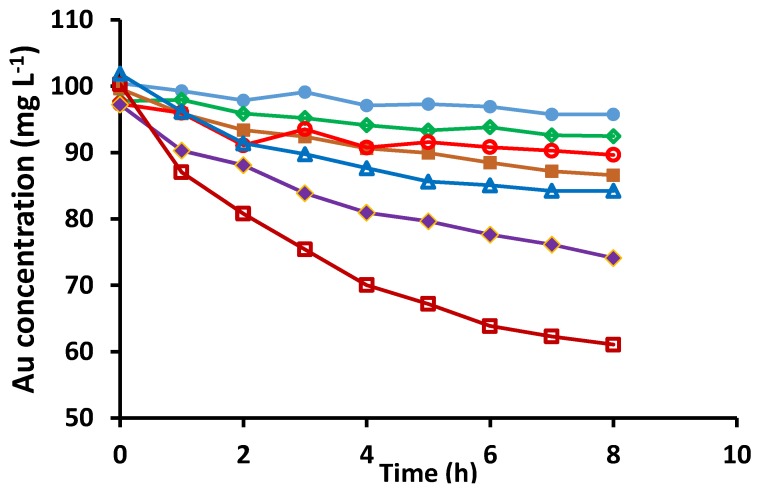
The extraction of Au(III) from 2.5 mol L^−1^ HCl using the following PIMs containing PVC, 1-dodecanol and Cyphos^®^ IL 104 (IL), and characterized as “clear” in Table 1: ● 20 wt% IL; ◇ 20 wt% IL and 2.4 wt% 1-dodecanol; ■ 20 wt% IL and 5 wt% 1-dodecanol; ○ 25 wt% IL; ◆ 25 wt% IL and 2.5 wt% 1-dodecanol; □ 25 wt% IL and 5 wt% 1-dodecanol; and △ 30 wt% IL (The remaining percentage in all PIMs is PVC). Aqueous phase: 100 mL, 100 mg L^−1^ Au(III), 2.5 mol L^−1^ HCl; membrane mass and thickness: 60 ± 3 mg, 50 ± 5 µm; shaking rate: 150 rpm. Data points are the average of 3 extraction experiments with an average standard deviation of 0.64 mg L^−1^. Membranes of similar masses and sizes were used.

PIMs that were homogeneous, transparent, and flexible (listed in Table 1 as “clear”) were tested for their ability to extract Au(III) from 2.5 mol L^−1^ HCl, and the results are shown in Figure 1, as the decrease in the Au(III) concentration in the aqueous phase with time. Figure 1 demonstrates a considerably higher extraction rate, and amount extracted for PIMs with 1-dodecanol as the modifier than for PIMs without it. The PIM composition containing 25 wt% Cyphos^®^ IL 104, 5 wt% 1-dodecanol and 70 wt% PVC, corresponding to the highest extraction rate and amount of Au(III) extracted (Figure 1), was selected as the optimum PIM composition.

**Table 1:** Homogeneity of polymer inclusion membranes (PIMs) containing poly(vinyl chloride) (PVC), Cyphos^®^ IL 104 and 1-dodecanol.

		Cyphos^®^ IL 104 (wt%)			
		**20.0**	**25.0**	**30.0**	**40.0**
**1-Dodecanol (wt%)**	**0.0**	**Clear**	**Clear**	**Clear**	Cloudy
	**2.5**	**Clear**	**Clear**	Cloudy	Oily surface
	**5.0**	**Clear**	**Clear**	Cloudy	Oily surface
	**7.5**	Oily surface	Oily surface	Oily surface	Oily surface
	**10.0**	Oily surface	Oily surface	Oily surface	Oily surface
	**12.5**	Oily surface	Oily surface	Oily surface	Oily surface

### 2.2. Effect of the HCl Concentration

**Figure 2:**
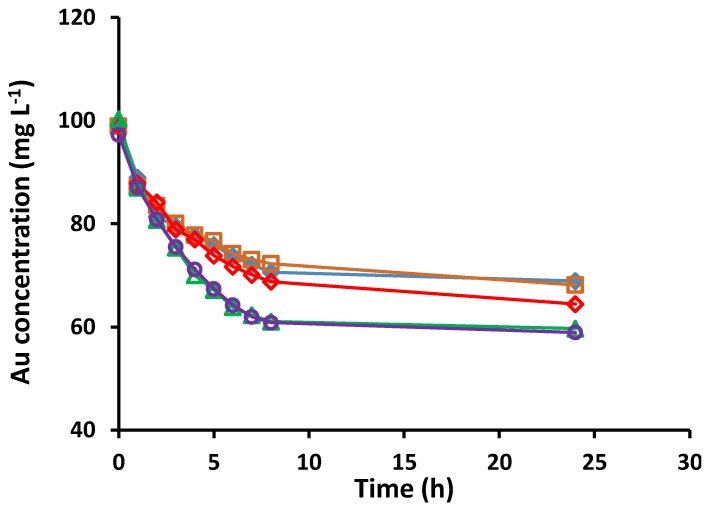
Extraction of Au(III) from aqueous solutions with different HCl concentrations (◆ 0.05, □ 0.10, ⃟ 1.0, △ 2.5, and ◯ 3.0 mol L^−1^) (Aqueous phase: 100 mL, 100 mg L^−1^ Au(III); membrane mass and thickness: 60 ± 3 mg, 50 ± 5 µm; membrane composition: 25 wt% Cyphos^®^ IL 104, 5 wt% 1-dodecanol and 70 wt% PVC; shaking rate: 150 rpm). Data points are the average of 3 extraction experiments with an average standard deviation of 0.59 mg L^−1^. Membranes of similar masses and sizes were used.

The effect of the concentration of HCl in the range of 0.050–3.0 mol L^−1^ on Au(III) extraction was studied. The extraction results in Figure 2 show an increase in the Au(III) extraction rate and equilibrium Au(III) amount extracted, as the concentration of HCl is increased from 0.050 mol L^−1^ to 2.5 mol L^−1^. As shown in Section 2.3, the extraction mechanism involves the positively charged phosphonium group of Cyphos^®^ IL 104, forming an ion-pair with the tetrachloroaurate(III) anion, which is accompanied by the formation of an ion-pair between the H^+^ ion and the negatively charged phosphinate group. Therefore, an increase in the concentration of HCl should lead to an increase in the extraction rate and the amount of Au(III) extracted. This effect was observed for HCl concentrations lower than 2.5 mol L^−1^ but there was no improvement in the rate of Au(III) extraction and the amount extracted when the concentration of HCl was higher than this value. Hence, 2.5 mol L^−1^ was chosen as the optimum HCl concentration.

### 2.3. The Au(III) Extraction Mechanism

In order to elucidate the Au(III) extraction mechanism, it is important first to establish the stoichiometry of the extracted Au(III)-Cyphos^®^ IL 104 adduct. This was carried out using a variation of the method described by St. John *et al*. [10]. This involved extracting Au(III) from 2.5 mol L^−1^ HCl using PIM segments of different masses (PIM composition, 25 wt% Cyphos^®^ IL 104, 5 wt% 1-dodecanol and 70 wt% PVC). The masses of the PIM segments were varied to achieve different mole ratios between the extractant in the PIM to the Au(III) in the solution. 

**Figure 3:**
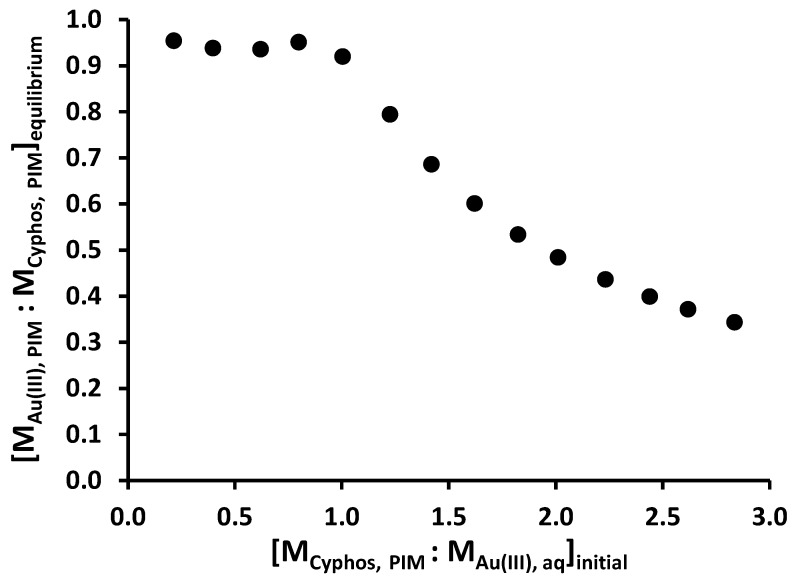
Determination of the stoichiometry of the Au(III)-Cyphos® IL 104 adduct (Aqueous solution: 50 mL, 50 mg L^−1^ Au(III) in 2.5 mol L^−1^ HCl; membrane composition: 25 wt% Cyphos^®^ IL 104, 5 wt% 1-dodecanol and 70 wt% PVC; shaking rate: 150 rpm). Data points are the average of 3 extraction experiments with an average standard deviation of 0.14 mg L^−1^. Membranes of similar masses and sizes were used.

The results are shown in Figure 3 as a plot of the mole ratio of Au(III) to Cyphos^®^ IL 104 in the PIM at equilibrium ([M_Au(III), PIM_ : M_Cyphos,_ _PIM_]_equilibrium_) *versus* the initial mole ratio of Cyphos^®^ IL 104 in the PIM to Au(III) in the solution ([M_Cyphos,_ _PIM_ : M_Au(III), aq_]_equilibrium_). At an initial mole ratio of Cyphos^®^ IL 104 in the PIM to Au(III) in a solution of less than 1:1, the equilibrium mole ratio of Au(III) to Cyphos^®^ IL 104 in the PIM is constant with a value of approximately 1:1. This corresponds to a “full loading” of the available Cyphos^®^ IL 104 in the PIM. At mole ratios of Cyphos^®^ IL 104 to Au(III) in the solution of greater than 1:1, there is insufficient Au(III) extracted to “fully load” Cyphos^®^ IL 104, and so the equilibrium mole ratio of Au(III) to Cyphos^®^ IL 104 in the PIM decreases. This result suggests that the stoichiometry of the adduct formed in the PIM between Cyphos^®^ IL 104 and Au(III) is 1:1, and, together with a decrease in the pH of the receiver phase during back-extraction (see Section 2.4, Equation (2)), supports an extraction mechanism described by Equation (1). The membrane loading capacity of the PVC-based PIM was determined in these experiments as 0.38 meq g^-1^.

[P]^+^ [PO_2_]^−^_(PIM)_ + HAuCl_4(aq)_ → [P]^+^ [AuCl_4_]^−^ H^+^ [PO_2_]^−^_(PIM)_ (1)

where [P]^+^ and [PO_2_]^−^ represent trihexyl(tetradecyl)phosphonium and bis(2,4,4-trimethylpentyl) phosphinate ions, respectively, and subscripts PIM and aq refer to the PIM and aqueous phases, respectively.

### 2.4. Au(III) Back-Extraction

The back-extraction of Au(III) from the PIM was studied using 0.10 mol L^−1^ solutions of various back-extraction reagents and the results are summarized in Table 2. The results show that Na_2_SO_3_ is the most successful back-extraction reagent, giving almost complete back-extraction of Au(III) and providing a high initial flux. This can be explained by the reducing power of Na_2_SO_3_, which can reduce Au(III) to Au(I), which then forms the complex [Au(SO_3_)_2_]^3−^ in the receiver solution. This triply-charged anion is less favorably re-extracted into the membrane due to its size and charge. A decrease in the pH of the receiver solution was also observed allowing the back-extraction process to be represented by Equation (2):

[P]^+^ [AuCl_4_]^−^ H^+^ [PO_2_]^−^_(PIM)_ + 3SO_3_^2−^_(aq)_ + H_2_O_(aq)_→ [P]^+^ [PO_2_]^-^_(PIM)_ + [Au(SO_3_)_2_]^3-^_(aq)_ + 3H^+^_(aq)_ + 4Cl^-^_(aq)_ + SO_4_^2-^_(aq)_ (2)

**Table 2:** The percentage back-extraction of Au(III) after 35 h, and the initial back-extraction flux.

Stripping reagent	%Back-extraction	Initial flux (mol m^−2^ s^−1^)
NaCl	13.7	1.26 × 10^−3^
HCl	18.9	1.71 ×10^−3^
KNO_3_	13.8	7.64 × 10^−4^
HNO_3_	8.25	1.28 × 10^−3^
KSCN	8.71	7.85 × 10^−4^
Na_2_SO_3_	95.4	2.60 × 10^−3^
Na_2_S_2_O_3_	68.2	2.63 × 10^−3^
Thiourea	76.1	1.61 × 10^−3^
Na_2_SO_4_	17.2	1.78 × 10^−3^
H_2_SO_4_	0.00	0.00
NaClO_4_	58.0	6.50 × 10^−4^
KBr	11.9	8.42 × 10^−4^

Note: Values are the average of 3 back-extraction experiments conducted for each back-extraction reagent with an average relative standard deviation of the % back-extraction of 1.67% and of the initial flux of 7.23 × 10^−5^ mol m^−2^ s^−1^. Membranes of similar masses and sizes were used. The PIMs were loaded with Au(III) using 100 mL of a 100 mg L^−1^ Au(III) solution in 2.5 mol L^−1^ HCl.

### 2.5. Effect of pH of the Receiver Solution

**Figure 4:**
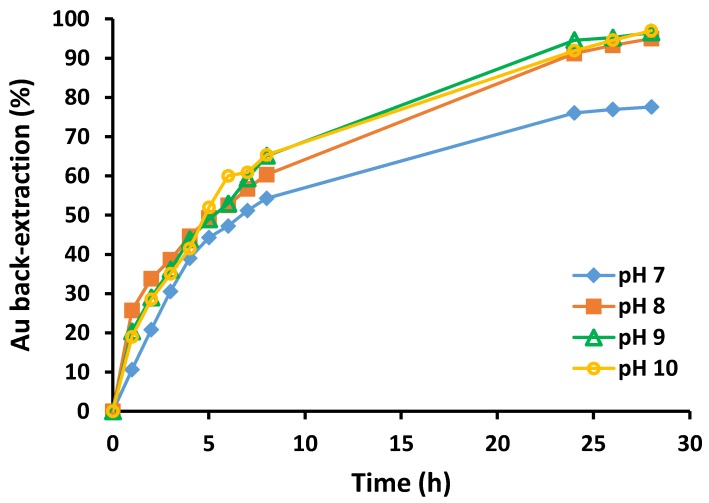
Percentage back-extraction of Au(III) with Na_2_SO_3_ *vs.* time for different pH values. Receiver solution volume and composition: 100 mL, 0.10 mol L^−1^ Na_2_SO_3_; membrane mass and thickness: 60 ± 3 mg, 50 ± 5 µm; membrane composition: 25 wt% Cyphos^®^ IL 104, 5 wt% 1-dodecanol and 70 wt% PVC; shaking rate: 150 rpm. Data points are the average of 3 extraction experiments with an average standard deviation of 1.24 mg L^-1^. Membranes of similar masses and sizes were used.

Minsker *et al.* [11] have reported that PVC-based PIMs are prone to dehydrochlorination in solutions with high pH values, but are stable in weakly alkaline solutions [12]. Additionally, the speciation of sulfite is affected by the pH of the receiver solution [13]. The effect of the pH of the Na_2_SO_3_ solution on the back-extraction of Au(III) was studied in the region of pH 7–10 by adjusting the pH using HCl or NaOH solutions, and the results are shown in Figure 4. The percentage back-extraction of Au(III) at pH 7 was lower than that at the higher pH values, and this could be explained by the lower mole fraction of the SO_3_^2−^ species present at pH 7 (0.398) compared to pH 8 (0.869), pH 9 (0.985), and pH 10 (0.998) [13]. The membranes exposed to Na_2_SO_3_ solutions at pH 9 or 10 during the back-extraction process showed the presence of surface black spots, which were probably caused by localized dehydrochlorination of PVC in alkaline media. However, at pH 7 and pH 8, the membranes appeared visually to be unchanged. In addition, very small differences in both the rate and percentage back-extraction of Au(III) were observed at pH 8 and higher. Therefore, pH 8 was chosen as the optimal pH for Au(III) back-extraction without observing any effects of dehydrochlorination of PVC.

### 2.6. Effect of the Na_2_SO_3_ Concentration of the Receiver Solution

**Figure 5:**
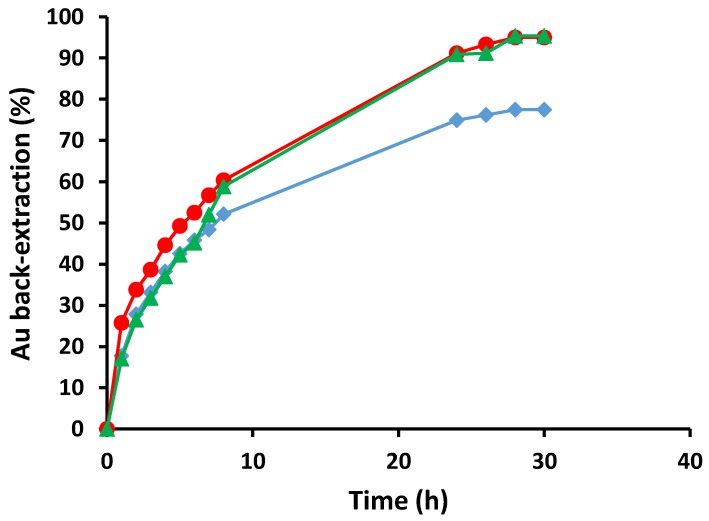
The percentage back-extraction of Au(III) *vs.* time for different concentrations of Na_2_SO_3_ (◆ 0.05, ● 0.10 and ▲ 1.0 mol L^−1^) (Receiver solution: 100 mL, pH 8; membrane mass and thickness: 60 ± 3 mg, 50 ± 5 µm; membrane composition: 25 wt% Cyphos^®^ IL 104, 5 wt% 1-dodecanol and 70 wt% PVC; shaking rate: 150 rpm). Data points are the average of 3 extraction experiments with an average standard deviation of 1.30 mg L^−1^. Membranes of similar masses and sizes were used.

The effect of the concentration of Na_2_SO_3_ on the back-extraction of Au(III) was studied using 0.05, 0.10 and 1.0 mol L^−1^ Na_2_SO_3_ receiver solutions at pH 8 and the results are shown in Figure 5. The 0.05 mol L^−1^ Na_2_SO_3_ solution was only able to back-extract close to 80% of the Au(III) present in the Au(III) loaded membrane, while both 0.10 mol L^−1^ and 1.0 mol L^−1^ Na_2_SO_3_ solutions back-extracted 95% of the Au(III). Therefore, 0.10 mol L^−1^ was chosen as the preferred concentration of Na_2_SO_3_ in the receiver solution.

### 2.7. Repeated Extraction/Back-Extraction Cycles

Three consecutive extraction/back-extraction cycles were carried out with the same membrane. It was found that after the first cycle, the membrane lost its extraction ability. Additionally, after the first back-extraction, a significant amount of a white film formed on the surface of the PIM, which was verified by gas chromatography to contain 1-dodecanol. It appears that the loss of 1-dodecanol and the formation of the 1-dodecanol layer on the membrane surface are the reasons for the decline in the performance of the PIM.

## 3. Experimental Section

### 3.1. Materials

**Scheme 1:**
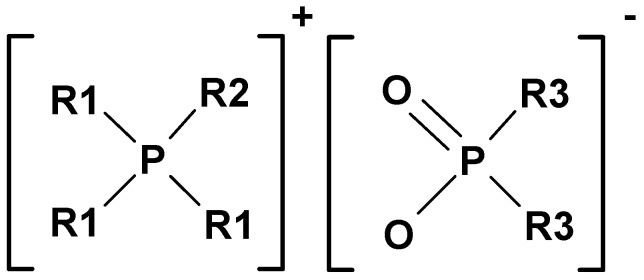
Chemical structure of Cyphos^®^ IL 104 ([P]^+^ [PO_2_]^−^) where R1 = hexyl, R2 = tetradecyl and R3 = trimethylpentyl.

Cyphos^®^ IL 104 (trihexyl(tetradecyl)phosphonium bis(2,4,4-trimethylpentyl) phosphinate) (Strem Chemicals Inc., min. 95%) (Scheme 1) was used as received. High molecular weight powdered PVC (MM=80,000, Fluka) was used as the base polymer in the membrane preparation. Tetrahydrofuran (Chem-supply, Australia) was used as received. The following modifiers and plasticizers used in the PIM compositions were used as received: 1-dodecanol (Aldrich), 1-tetradecanol (Aldrich), 2-nitrophenol octyl ether (Fluka), dioctyl phthalate (Aldrich), and tri-2-ethyl-hexyl phosphate (Aldrich). Au(III) standards were prepared from a 1000 mg L^−1^ Au standard (BDH Spectrosol) by dilution with hydrochloric acid (BDH AnalR). Au(III) solutions were prepared from chloroauric acid (HAuCl_4_) (Aldrich) by dissolving it in hydrochloric acid solutions. In the Au(III) back-extraction studies, reagents were prepared by dissolving thiourea (BDH), sodium chloride (Chem-supply), potassium thiocyanate (BDH), sodium perchlorate (Chem-supply), sodium nitrate (Ajax), nitric acid (BDH AnalR), sulfuric acid (BDH AnalR), sodium thiosulfate (Ajax), sodium sulfite (BDH AnalR), sodium sulfate (BDH AnalR), or potassium bromide (Chem-supply) in deionized water (18 MΩ cm, Millipore, Synergy 185, France). The pH of the receiver solutions was adjusted by using hydrochloric acid or sodium hydroxide (Chem-supply) solutions. 

### 3.2. Apparatus

Au(III) concentrations were determined using atomic absorption spectrometry (AAS) (Hitachi Z-2000 Series Polarized Zeeman atomic absorption spectrophotometer, Japan). Au(III) extraction and back-extraction experiments were conducted using an orbital shaker (Platform Mixer OM6, Ratek). Optical microscopy and membrane thickness measurements were conducted with a Motic SMZ-140 stereo microscope (Motic, China) with 60× magnification in combination with a MoticCam 1000 microscope camera (Motic, China). 

A gas chromatograph (Model GC 2010, Shimadzu) equipped with an EC-Wax capillary column (30 cm × 0.25 mm × 0.25 µm, Alltech) and a flame ionization detector was used in the determination of 1-dodecanol. 

### 3.3. Membrane Preparation

Cyphos^®^ IL 104, PVC and a plasticizer or modifier (when required), with a total mass of 400 mg, were dissolved in a small volume (8–10 mL) of THF. The solution was poured into a glass ring with a diameter of 7.5 cm, positioned on a flat glass plate. The mixture was covered with a filter paper and a watch glass to allow slow evaporation of the solvent over a 24 h period leaving a transparent and flexible circular membrane. The membrane was removed from the glass plate and cut to size (mass: 60 ± 3 mg, diameter: 3.5 cm, thickness: 50 ± 5 μm).

### 3.4. Membrane Extraction and Back-Extraction Experiments

In the extraction experiments membranes were immersed in 100 mL of 100 mg L^−1^ Au(III) solutions containing 2.5 mol L^−1^ HCl in glass jars which were shaken on an orbital shaker at 150 rpm. Samples of the Au(III) solution (0.2 mL) were removed at predetermined time intervals throughout the course of the experiments. The samples were diluted to 4 mL with deionized water and the Au(III) concentration was determined by AAS.

Membranes containing Au(III) were back-extracted by immersing them in receiver solutions (100 mL) containing a stripping reagent, which were shaken on an orbital shaker at 150 rpm. The back-extraction process was monitored by continuously sampling the solutions (0.2 mL) at predetermined time intervals as in the extraction experiments.

### 3.5. Stoichiometry of the Au(III)/Cyphos^®^ IL 104 Adduct

The stoichiometry of the extracted adduct was determined by using a variation of the method described by St. John *et al.* [10]. Individual PIM segments of composition 25 wt% Cyphos^®^ IL 104, 5 wt% 1-dodecanol and 70 wt% PVC with varying masses were immersed in individual jars containing 50 mL of a solution containing 50 mg L^−1^ Au(III) and 2.5 mol L^−1^ HCl. The masses of the membrane segments were varied between 8 and 125 mg, which allowed the variation of the mole ratio of Cyphos^®^ IL 104 in the PIM to Au(III) in the solution between 0.20:1 to 3.2:1. The jars were agitated on an orbital shaker at 150 rpm for 5 days to allow the systems to reach equilibrium and the Au(III) concentration in each jar was determined by AAS.

### 3.6. Initial Flux Calculations

The initial flux (*J_0_*, mol m−2 s−1) was calculated according to Fick’s first law, by fitting the transient Au(III) concentration with an exponential decay function ($\bar{C}=a_{1}+a_{2}e^{-a_{3}t}$) the first derivative of which (${\left(d\bar{C}/dt\right)}_{t=0}$) was used to calculate *J_0_* according to Equation (3):

(3) $$J_{0}=\frac{V}{S}\cdot {\left[\frac{dC}{dt}\right]}_{t=0}$$

where *V* is the volume of the solution (m^3^), *S* is the surface area of the membrane (m^2^), C is the concentration of Au(III) (mol m^−3^), and *t* is time (s).

## 4. Conclusions

This study examined a number of PIMs composed of Cyphos^®^ IL 104 as the extractant for Au(III) with PVC as the base polymer. Some of the PIMs also incorporated a plasticizer or modifier. Only PIMs prepared without a plasticizer or modifier or with 1-dodecanol as the modifier were homogeneous, transparent and flexible. The other membranes had oily surfaces, which indicated incompatibility of the components in the PIMs.

The membrane with a composition of 25 wt% Cyphos^®^ IL 104, 5 wt% 1-dodecanol and 70 %wt PVC showed the fastest rate of extraction and highest amount of Au(III) extracted from solutions with a HCl concentration of 2.5 mol L^−1^. The stoichiometry of the adduct formed between Cyphos^®^ IL 104 and Au(III) in the PIM was determined to be [P]^+^[AuCl_4_]^−^H^+^[PO_2_]^−^, where [P]^+^ and [PO_2_]^−^ represent trihexyl(tetradecyl)phosphonium and bis(2,4,4-trimethylpentyl) phosphinate ions, respectively.

An extensive study of a number of back-extracting reagents for the back-extraction of Au(III) from Au(III) loaded PIMs found that the solution containing 0.10 mol L^−1^ Na_2_SO_3_ at pH 8 gave the highest percentage back-extraction and the highest initial back-extraction flux. The mechanism for back-extraction is suggested to involve reduction of Au(III) to Au(I), with the formation of the complex [Au(SO_3_)_2_]^3^^−^ in the receiver aqueous solution. 

The loss of 1-dodecanol from the PIM after one extraction/back-extraction cycle and the formation of the 1-dodecanol layer on the membrane surface resulted in deterioration of the performance of the PIM. This characteristic inhibits the use of the PIM in a continuous transport system unless another modifier can be found to eliminate the problem. Nevertheless, the newly developed PIM could be potentially suitable for a single use process for efficiently recovering Au(III) from diluted aqua regia solutions of electronic scrap or recycled jewelry. The high price of gold justifies financially this approach.
